# Prevalence and determinants of chronic respiratory diseases in adults in rural Sudan

**DOI:** 10.5588/ijtld.22.0655

**Published:** 2023-11-01

**Authors:** R. Ahmed, R. Osman, R. Nightingale, D. Nagem, R. Thomson, R. Malmborg, M. Elmustafa, A. F. S. Amaral, J. Patel, P. Burney, A. El Sony, K. Mortimer

**Affiliations:** 1 The Epidemiological Laboratory, Khartoum, Sudan; 2 Liverpool School of Tropical Medicine, Liverpool, UK; 3 Nordic Innovation, Oslo, Norway; 4 University of Gezira, Wad Medani; 5 Wad Medani College of Medical Sciences and Technology, Wad Medani, Sudan; 6 National Heart and Lung Institute, Imperial College London, London; 7 Liverpool University Hospitals NHS Foundation Trust, Liverpool; 8 University of Cambridge, Cambridge, UK; 9 School of Clinical Medicine, College of Health Sciences, University of KwaZulu-Natal, Durban, South Africa

**Keywords:** CRD, CAO, COPD, asthma, low FVC

## Abstract

**BACKGROUND:**

Chronic respiratory diseases (CRDs) greatly contribute to worldwide mortality. Despite new data emerging from Africa, prevalence estimates and determinants of CRDs in rural settings are limited. This study sought to extend the existing research conducted in urban Sudan by conducting a rural comparison.

**METHODS:**

Participants aged ≥18 years (*n* = 1,850), living in rural Gezira State completed pre-and post-bronchodilator spirometry and a questionnaire. Prevalence of respiratory symptoms and spirometric abnormalities were reported. Regression analyses were used to identify risk factors for CRDs.

**RESULTS:**

Prevalence of chronic airflow obstruction (CAO) was 4.1% overall and 5.5% in those aged ≥40 years. Reversibility was seen in 6.4%. Low forced vital capacity (FVC) was seen in 58.5%, and at least one respiratory symptom was present in 40.7% of the participants. CAO was more common among people aged 60–69 years (OR 2.07, 95% CI 1.13–3.82) and less common among highly educated participants (OR 0.50, 95% CI 0.27–0.93). Being underweight was associated with lower FVC (OR 3.07, 95% CI 2.24–4.20).

**CONCLUSIONS:**

A substantial burden of CRD exists among adults in rural Sudan. Investment in CRD prevention and management strategies is needed.

## Abstract (fr)

**CONTEXTE:**

Les maladies respiratoires chroniques (CRD) contribuent grandement à la mortalité mondiale. Malgré l’apparition de nouvelles données en Afrique, les estimations de la prévalence et les déterminants des maladies respiratoires chroniques en milieu rural sont limités. Cette étude visait à étendre les recherches existantes menées dans les zones urbaines du Soudan en effectuant une comparaison avec les zones rurales.

**MÉTHODES:**

Les participants âgés de ≥18 ans (n = 1 850), vivant dans l'État rural de Gezira, ont effectué une spirométrie pré et post-bronchodilatateur et répondu à un questionnaire. La prévalence des symptômes respiratoires et des anomalies spirométriques a été rapportée. Des analyses de régression ont été utilisées pour identifier les facteurs de risque des maladies respiratoires chroniques.

**RÉSULTATS:**

La prévalence de l’obstruction chronique du flux d’air (CAO) était de 4,1% dans l’ensemble et de 5,5% chez les personnes âgées de ≥40 ans. La réversibilité a été observée dans 6,4% des cas. Une faible capacité vitale forcée (FVC) a été observée chez 58,5% des participants et au moins un symptôme respiratoire était présent chez 40,7% d’entre eux. La CAO était plus fréquent chez les personnes âgées de 60 à 69 ans (OR 2,07; IC 95% 1,13–3,82) et moins fréquent chez les participants ayant un niveau d'éducation élevé (OR 0,50; IC 95% 0,27–0,93). L’insuffisance pondérale était associée à une FVC plus faible (OR 3,07; IC 95% 2,24–4,20).

**CONCLUSIONS:**

La charge de morbidité chronique est importante chez les adultes des zones rurales du Soudan. Il est nécessaire d’investir dans des stratégies de prévention et de gestion de la CRD.

Although the global burden of chronic respiratory diseases (CRDs) such as chronic obstructive pulmonary disease (COPD) and asthma is high and expected to increase,^1^,^2^ they receive limited attention.^2^ The Global Burden of Disease study estimated that COPD affected almost 212 million adults in 2019 and resulted in 3 million deaths, with 62.6% of the burden of COPD and lung cancer occurring in low- and middle-income countries (LMICs).^2^,^3^ COPD is poorly recognised and undertreated in most populations.^4^ Asthma affected 262 million people and resulted in almost half a million deaths in 2019.^1^ Asthma-related deaths in LMICs account for 96% of global asthma-related deaths and 84% of global disability-adjusted life-years (DALYs).^1^,^3^

Data about CRDs in sub-Saharan Africa (SSA) and the Middle East and North Africa (MENA) regions are scarce, but suggest the burden is high.^4^ High prevalence of chronic airflow obstruction (CAO) has been documented across the region,^5^ with spirometric obstruction seen in 10% of urban Sudanese adults in a recent study.^6^ The prevalence and underlying causes of low forced vital capacity (FVC) in SSA remain poorly understood;^7^ nonetheless, its association with mortality is well-documented.^8^ TB, early childhood infection, low body mass index, biomass fuel use, smoking and poverty are believed to be associated with CRDs in LMICs.^2^,^9^–^11^

Data on the prevalence and determinants of COPD and asthma in Sudan are scarce, although asthma is the third most frequent cause of hospitalisation and the number of asthma-related emergency admissions is increasing.^12^ Moreover, a high burden of uncontrolled asthma exists even in tertiary level hospitals in Khartoum, Sudan.^13^ The Burden of Obstructive Lung Disease (BOLD) Initiative developed standardised methods for estimating the burden and determinants of CAO in populations aged ≥40 years.^14^,^15^ Following an urban study in Khartoum State,^6^ we conducted a population-based cross-sectional (BOLD) study in rural Gezira State, Sudan, to explore the prevalence and determinants of CRD symptoms and spirometric abnormalities among adults in rural Sudan.

## METHODS

### Setting

Gezira State is situated in central Sudan, and has an estimated population of 3,780,915, most of which (80.4%) live in rural areas.^16^ This population is distributed across seven localities, 40 administrative units and 689 villages.

### Sampling

Three localities were randomly selected for inclusion in this study. Thirty-five villages were randomly selected and 30 households per village were included. Households included nomadic and Cambo (rural, mixed-composition, displaced community) families present at the time of mapping and sampling.^17^

### Participants

Using the BOLD protocol,^15^ participants aged ≥18 years living in rural Gezira State were included. We excluded institutionalised people, the medically unfit and pregnant women in their last trimester.

### Data collection and management

All participants completed a structured interview in the local Arabic language administered by trained interviewers. Anthropometric measurements, pre-bronchodilator (pre-BD) and post-BD spirometry data were collected following American Thoracic Society (ATS) guidelines using the Easy One system (ndd Medizintechnik, Zurich, Switzerland) by three trained certified technicians.^15^ A basic information or refusal questionnaire was filled out for those not willing to participate in the full study. The clinical data obtained included height, weight, pulse rate, and waist and hip circumference. Quality control was done at the BOLD coordinating centre. Usable spirometry was defined as two or more acceptable blows, with forced expiratory volume in 1 sec (FEV_1_) and FVC repeatability within 200 mL. Acceptable manoeuvres were defined as those with a rapid start (back-extrapolated volume, 150 mL or 5% of the FVC), lack of cough during the first second and a small end-of-test volume (<40 mL during the final second). Calibration of all spirometers was verified to be accurate within 3.0% using a 3.00 L syringe at the beginning of each day of testing. Spirometry traces were then classified according to FEV_1_/FVC < lower limit of normality (LLN).

### Statistical analysis

Prevalence estimates of spirometric abnormalities stratified by age and sex were reported using the US National Health and Nutrition Examination Study III (NHANES III) reference equations for White Americans (Caucasians).^15^ CAO Stage 1 or higher was defined by the LLN for post-BD FEV_1_/FVC, based on the NHANES III Caucasian reference equations for age and sex.^18^ Reversibility was defined using ATS and European Respiratory Society guidelines; difference in FEV_1_ (post BD minus pre-BD) ≥200 mL and percentage change in FEV_1_ ≥12%. Prevalence estimates using locally derived spirometry values were also reported. These were derived from non-smoking Sudanese adults with no respiratory symptoms or diagnoses in the previous urban study.^6^

Univariable and multivariable logistic regression analyses were used to test associations between spirometry abnormalities and exposure variables, including age, sex, body mass index, education level, smoking status, smoking pack-years, exposure to indoor smoke from biomass fuel, occupation, and self-reported history of TB, hypertension, diabetes, and heart disease. A wealth score based on household asset ownership was used as a proxy for socio-economic status.^9^,^19^

Multivariable logistic regression models including sex, age and all variables from the univariable analysis with a* P* < 0.2 were developed. Prevalence of respiratory symptoms was reported and associations with study variables were tested using regression analysis. Associations between abnormal spirometry and respiratory symptoms were reported. Data were analysed using Stata IC v15 (StataCorp, College Station, TX, USA). Prevalence estimates and regression models were developed using survey weighting with the *Svy* package in Stata v15. To allow comparisons with the previous urban study, age groups were stratified to below and above 40 years.

### Ethical considerations

Written informed consent was obtained from study participants before data collection. Ethical approval was obtained from the Imperial College London, London, UK; the Ethics Committee at Gezira State Ministry of Health, Wad Medani, Sudan; and Liverpool School of Tropical Medicine, Liverpool, UK (11.03RS).

## RESULTS

**Figure 1:**
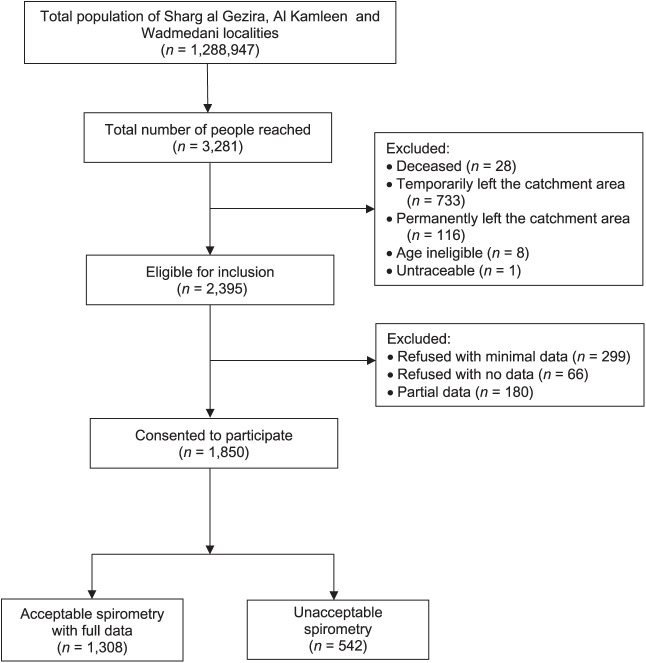
Participant flow diagram.

Between August 2015 and December 2016, 3,281 participants from 35 villages in Gezira were approached and invited to participate in the study. Of these, 2,395 were eligible for inclusion and 1,850 (response rate 56.3%) consented to take part. Questionnaires and spirometry were completed to the required standard by 1,308 participants (Figure 1).

### Participant characteristics

The mean age was 39.3 years, and 50% were women. Participants aged ≥40 years represented 45% of all participants. Almost 11% were current smokers. About 44% had high school or above education, with younger participants (<40 years) being more educated than older groups (66% vs. 36% higher education attainment, respectively). Overall, 17.3% of participants owned 6 of the 10 considered assets used in the construction of the wealth score, and 92% owned their own home. The average number of members in a household was 7. Farming was the most common occupation (39%), with 15% of farmers reporting more than 5 years of activity; 48% of participants had exposure to biomass fuels for more than 6 months in their lives. Moreover, 47% of participants had a normal body mass index (BMI). Hypertension was self-reported by 6.4% of participants (70% were women), followed by diabetes (3.3%, of whom 57% were women). Both heart disease and TB were reported by 0.3% (Table 1).

**Table 1:** Characteristics of all participants who completed a full BOLD core questionnaire, including those with and without spirometry results by age groups*

Variable	Total *n* (%)	18–39 years *n* (%)	≥40 years *n* (%)
Age group, years (*n* = 1,844)		1,013 (55)	831 (45)
18–29	626 (34)		
30–39	387 (21)		
40–49	328 (17.8)		
50–59	240 (13.0)		
60–69	173 (9.4)		
≥70	90 (4.9)		
Sex (*n* = 1,850)			
Male	924 (50)	486 (48)	434 (52)
Female	926 (50)	527 (52)	397 (48)
Level of education (*n* = 1,804)			
None	398 (22.1)	101 (10.2)	297 (36.9)
Primary school	483 (26.8)	250 (25.2)	231 (28.7)
Middle school	124 (6.9)	40 (4)	84 (10.5)
High school or above	799 (44.3)	603 (60.7)	192 (23.9)
Home ownership (*n* = 1,831)			
Yes	1,679 (91.7)	912 (91.3)	761 (92.1)
No	151 (8.3)	86 (8.6)	65 (7.9)
People in households (*n* = 1,832)			
<5	290 (15.9)	134 (13.4)	156 (18.9)
5–9	1,163 (63.5)	670 (67.1)	488 (59.0)
≥10	379 (20.7)	195 (19.5)	183 (22)
Smoking status (*n* = 1,833)			
Current smoker	197 (10.8)	141 (14.1)	54 (6.5)
Ex-smoker	191 (10.4)	56 (5.6)	135(16.3)
Never smoked	1,445 (78.8)	803 (80.3)	638 (77.2)
Pack-years of smoking (*n* = 1,809)			
Never smoked	1,445 (79.9)	803 (81)	638 (78.6)
>0 and <10	252 (13.9)	161 (16.3)	89 (11)
≥10	112 (6.2)	27 (2.7)	85 (10.5)
20 pack-years of smoking (*n* = 1,809)			
<20 years	1,764 (97.5)	986 (99.5)	772 (95.1)
≥20 years	45 (2.5)	5 (0.5)	40 (4.9)
Biomass fuel exposure (*n* = 1,814)			
Yes	876 (48.3)	383 (38.7)	491 (60)
No	938 (51.7)	607 (61.3)	327 (40)
Farm work for ≥3 months (*n* = 1,774)			
Yes	697 (39.3)	308 (31.8)	389 (48.6)
No	1,077 (60.7)	660 (68.2)	412 (51.4)
Body mass index, kg/m^2^ (*n* = 1,700)			
Underweight (<18.5)	173 (10.2)	139 (15)	34 (4.4)
Normal (18.5–24.9)	800 (47.1)	467 (50.4)	329 (42.8)
Overweight (25.0–29.9)	415 (24.4)	189 (20.4)	225 (29.3)
Obese (≥30)	312 (18.4)	131 (14.2)	181 (23.5)
Reported history of TB (*n* = 1,825)			
Yes	6 (0.3)	2 (0.2)	4 (0.5)
No	1,819 (99.7)	993 (99.8)	820 (99.5)
Reported history of hypertension (*n* =1,829)			
Yes	117 (6.4)	12 (1.2)	105 (12.7)
No	1,712 (93.6)	985 (98.8)	721 (87.3)
Reported history of diabetes (*n* = 1,828)			
Yes	60 (3.3)	5 (0.5)	55 (6.7)
No	1,768 (96.7)	991 (99.5)	771 (93.3)
Reported history of heart disease (*n* = 595)			
Yes	6 (0.3)	1 (0.1)	5 (0.6)
No	1,821 (99.7)	995 (99.9)	820 (99.4)
Current Mokken scale, mean ± SD *(n =* 1,824)	5.21 ± 2.36	5.4 ± 2.32	5.02 ± 2.04

* Data on the two age groups may not add up to the row total as these are based on data completeness of each variable for those who completed the full BOLD core questionnaire.

BOLD = Burden of Obstructive Lung Disease; SD = standard deviation.

### Respiratory symptoms

At least one respiratory symptom was reported by 40.7% of participants, with those over 70 years old showing the highest prevalence of symptoms (60.6%). Usual cough was the most frequently reported symptom (19.5%), with the highest prevalence recorded in participants aged 18–29 years (22.5%). Chronic cough (lasting for more than 3 months per year) was reported by 2.4% (standard error [SE] 0.02). Production of phlegm was reported by 14% (SE 1.9) and chronic production of phlegm was reported by 2.7% (SE 0.001). Shortness of breath was reported by 18.7%. Wheeze was the least commonly reported symptom (4.9%). Overall, females and participants over 70 were more symptomatic than other groups. Among participants with respiratory symptoms, 48.3% were ever smokers and 45.5% used biomass for ≥6 months. Participants aged ≥40 years showed higher prevalence of respiratory symptoms 42.2%. Medically diagnosed respiratory disease was reported by 7.4% of the participants. Asthma and COPD, whether or not diagnosed by a physician, were reported by respectively 6.4% and 7.4% of participants. Chronic bronchitis or emphysema was reported by 1.7%.

### Spirometry

**Figure 2:**
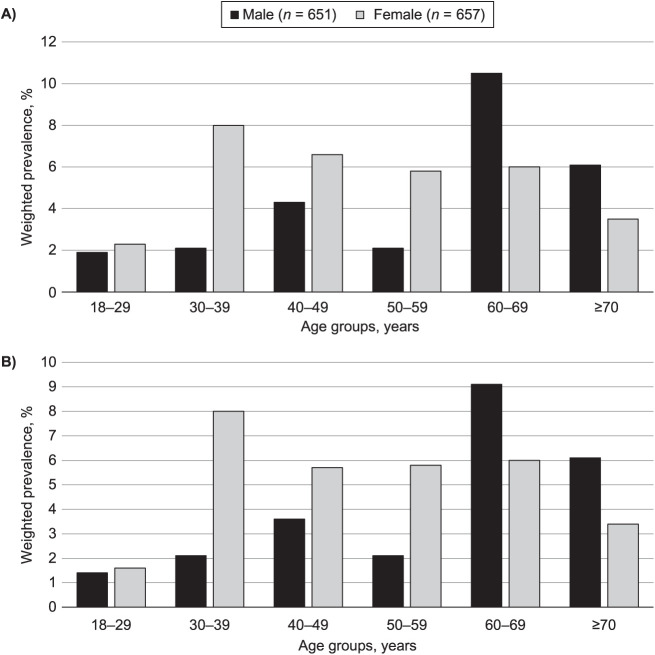
Estimated population prevalence of airway obstruction by age and sex using National Health and Nutrition Examination Survey reference ranges for the Sudanese population in participants completing standard American Thoracic Society spirometry (*n* = 1,308). **A)** Prevalence of Stage ≥1 CAO (post-BD FEV_1_/FVC < LLN); **B)** prevalence of Stage ≥2 CAO (post-BD FEV_1_/FVC < LLN and post-BD FEV_1_ <80% predicted). LLN = lower limit of normal; CAO = chronic airflow obstruction; BD = bronchodilator; FEV_1_ = forced expiratory volume in 1 sec; FVC = forced vital capacity.

The prevalence of CAO using NHANES III criteria was 4.1% (3.1% in men and 4.8% in women). The prevalence was about the same when using local reference equations (3.4% in men and 3.1% in women). Participants aged 60–69 years and participants who smoked over 20 years had the highest prevalence of Stage ≥1 and Stage ≥2 CAO (8.2%, SE 2.3 vs. 7.1%, SE 4.2; and 7.6%, SE 2.7 vs. 4.1%, SE 2.5, respectively) (Figure 2).

Participants aged ≥40 years had higher estimates of Stage ≥1 CAO (5.5%, SE 0.8; and 4.1%, SE 0.8) and CAO Stage ≥2 (5%, SE 0.6; and 2.7%, SE 0.5) using local reference range and NHANES III reference range, respectively. No association was identified between CAO and age using LLN.

For those with Stage ≥1 CAO, 53% (SE 0.09) reported at least one respiratory symptom that interfered with usual activities: casual cough (27%, SE 0.05), chronic cough (7.9%, SE 0.03), chronic phlegm (6.9%, SE 0.03), wheeze (9.8%, SE 0.04), shortness of breath (31%, SE 0.05) and breathing problems (25%, SE 0.00).

Using the NHANES III reference equations, the prevalence of low FVC (FVC < LLN), was 58.5% in both men and women. Using local reference equations, the prevalence was 5.3% in men and 9.1% in women. Participants in the younger group (18–39 years) had a higher prevalence of low FVC than those aged ≥40 years (61.5% vs. 52.6%).

Airway reversibility was present in 6.4%, and was more common in men (7.2%) than in women (5.7%). Airway reversibility scores were higher in those aged ≥40 years than in those aged 18–39 years (10% vs. 4.3%) in both men and women.

### Factors associated with respiratory symptoms

Years of education were significantly associated with having a cough (OR 1.02, 95% CI 1.00–1.04) in multivariable analysis. Females had the highest odds of having shortness of breath (OR 2.4, 95% CI 1.6–3.2) (Table 2). Phlegm production was associated with Stage ≥1 CAO using the local reference range (OR 1.1, 95% CI 1.0–1.2). Breathing problems that interfered with usual activities were associated with Stage ≥1 CAO based on LLN (OR 3.9, 95% CI 1.72–8.87). No significant difference was observed between age groups and respiratory symptoms.

**Table 2:** Multivariable associations of risk factors with Stage ≥1 CAO defined using the NHANES III reference range (post-BD FEV_1_/FVC < LLN; *n* = 61/1320) and with Stage ≥2 CAO defined using NHANES III reference range (post-BD FEV_1_/FVC < LLN and post-BD FEV_1_ < 80% predicted; *n *= 55/1320)

Variable	Multivariable association with CAO Stage ≥1		Multivariable association with CAO Stage ≥2	
	OR	95% CI	OR	95% CI
Age group, years				
18–29	1	—	1	—
30–39	2.07	0.59–7.35	2.99	0.57–15.74
40–49	2.53	0.32–19.79	3.01	0.17–53.70
50–59	1.25	0.12–12.83	2.28	0.31–16.73
60–69	3.65	0.73–18.24	4.93	0.84–28.81
≥70	1.7	0.12–24.35	2.78	0.19–40.16
Sex				
Male	1	—	1	—
Female	1.52	0.26–8.67	1.64	0.20–13.28
Level of education				
None	1	—	1	—
Primary school	1.4	0.03–59.02	1.21	0.33–4.45
Middle school	1.17	0.02–56.68	1.25	0.77–2.01
High school or above	1	0.01–157.5	1.03	0.48–2.18
Use of firewood in cooking >6 months				
No	1	—	1	—
Yes	0.61	0.09–3.96	0.64	0.17–2.38
Household used wood to heat water				
No	1	—	^†^	^†^
Yes	0.47	0.12–1.83	^†^	^†^

* *P *< 0.05.

^†^Not included in multivariable analysis.

CAO = chronic airflow obstruction; NHANES III = National Health and Nutrition Examination Survey III; BD = bronchodilator; FEV_1_ = forced expiratory volume in 1 sec; FVC = forced vital capacity; LLN = lower limit of normal; OR = odds ratio; CI = confidence interval.

### Factors associated with post-bronchodilator airway obstruction

Those aged 60–69 had the highest odds of having Stage ≥2 CAO using the local reference range (OR 2.07, 95% CI 1.13–3.82). In univariable analysis, participants with higher educational levels were less likely to have Stage ≥1 CAO (OR 0.50, 95% CI 0.27–0.93), as were those who used firewood for water heating (OR 0.32, 95% CI 0.13–0.77).

Participants aged ≥40 years had higher estimates of Stage ≥1 CAO (5.5%, SE 0.8; and 4.1%, SE 0.8) and Stage ≥2 (5%, SE 0.6; and 2.7%, SE 0.5) using local and NHANES III reference ranges, respectively. No association was identified between CAO and age using LLN.

### Factors associated with low FVC (FVC < LLN)

Being underweight had the highest odds of having low FVC using NHANES III reference ranges in both univariable and multivariable analysis (OR 2.25, 95% CI 1.13–5.64). Moreover, participants who had smoked for more than 20 pack-years were less likely to have low FVC in both univariable and multivariable analyses (OR 0.36, 95% CI 0.25–0.50). Participants aged ≥70 years were less likely to have low FVC in both univariable and multivariable analyses (OR 0.19, 95% CI 0.09–0.40; and OR 0.19, 95% CI 0.09–0.40) (Table 3). Using the local reference range, no association was identified between low FVC and any of the potential risk factors.

**Table 3:** Multivariable associations of risk factors with low FVC, defined using NHANES III reference range (FVC < LLN), *n* = 767/1320

Variable	Low FVC	
	OR	95% CI
Age group, years		
18–29	1	—
30–39	1.20	0.68–2.14
40–49	1.22	0.60–2.50
50–59	1.12	0.76–1.66
60–69	0.76	0.35–1.64
≥70	0.19*	0.09–0.40
Sex		
Male	1	—
Female	0.87	0.58–1.33
BMI, kg/m^2^		
Underweight (<18.5)	2.52*	1.127–5.64
Normal (18–25)	1	—
Overweight (25–30)	0.76	0.34–1.73
Obese (>30)	0.84	0.27–2.63
Years of education	0.98	0.84–1.14
Smoking pack–years		
0–10	1.91	0.35–10.44
10–20	3.18	0.13–73.20
≥20	^†^	^†^

* *P *< 0.05.

^†^No observation in this group.

FVC = forced vital capacity; NHANES III = National Health and Nutrition Examination Survey III; LLN = lower limit of normal; OR = odds ratio; CI = confidence interval; BMI = body mass index.

## DISCUSSION

Building on an earlier BOLD study conducted in urban Sudan,^6^ we conducted this population-based study in Gezira State to explore CRD symptoms and spirometric abnormalities in adults in rural Sudan.

The prevalence of CAO as indicated by persistent post-BD obstruction was 4.1%. Based on the LLN, in participants aged ≥40 years, the CAO prevalence was 5.5% and 3% using NHANES and local reference equations, respectively. These are lower than 10% and 5.7% from recent findings of an urban study in Sudan using NHANES and local reference equations, respectively.^6^

Using NHANES III reference equations shows a higher prevalence of obstruction compared to local reference equations, according to prior research.^8^ Local values may be more ethnically suitable than NHANES III despite being the only data available. However, as varying exposures in this environment might limit its application,^8^,^20^ using a standard reference when comparing prevalence across populations may be preferable.

The prevalence of CAO was higher than in other rural SSA settings, including Rwanda.^21^ These rates were however, far lower than those found in Uganda and rural Malawi.^22^,^23^ BOLD studies from countries in SSA such as South Africa^24^ and Nigeria,^25^ reported higher prevalence estimates compared to this study. However, similar studies from MENA countries, including Tunisia^26^ and Morocco,^27^ reported lower prevalence estimates, with the exception of Algeria.^28^

Age was the main risk factor for CAO in this study. This is consistent with the previous urban Sudan study and literature demonstrating that population ageing is one of the primary COPD risk factors, especially in developing countries with lower levels of smoking.^14^,^29^ The finding of those with high education level were less likely to have CAO is compatible with studies reporting an association of lower education with COPD.^30^

In line with the findings from the urban Sudan study, we did not observe any association between exposure to biomass fuel and obstruction. This is consistent with the 25 BOLD sites study which found no association between airflow obstruction and the use of solid fuels for cooking or heating.^31^ A recent review of studies on COPD and household air pollution concluded that it was not possible to define clear causal links between the two.^32^

In contrast to previous studies that have indicated a link between crowded housing and low socio-economic status with the progression of airflow limitation, our research reveals no such association between CAO and increased numbers of household inhabitants or low socio-economic status.^29^,^33^ These findings are consistent with the previous study in urban Sudan.^6^ A possible explanation for this discrepancy could be attributed to the unique nature of Sudanese housing, characterised by spacious living environments that may reduce the impact of exposure to household air pollution in these settings.

In this study, a high prevalence of respiratory symptoms was reported by 40% of the participants, with cough and shortness of breath being the most common symptoms. As expected, Stage ≥1 CAO was associated with breathing difficulties that interfered with daily activities. Moreover, respiratory symptoms were higher in those with Stage ≥1 CAO and breathing difficulty was the most common symptom among this group.

It is surprising to note that we found no association between respiratory symptoms and exposure to biomass fuel, even though 48% of the total participants were exposed to biomass fuels for ≥6 months, and females had the highest odds of having shortness of breath. This may be attributed to the higher prevalence of cough, shortness of breath and functional limitation due to breathing problems in the younger age group (18–39 years), 67% of which had no exposure to any biomass fuel. It is likely that the younger generation use gas and other cooking methods in these settings.

The absence of the association between respiratory symptoms and smoking might be explained by having 69% of those who ever smoked had less than 10 years of exposure. In addition, childhood respiratory infections may have contributed to this high prevalence, as pneumonia is the second highest cause of hospitalisation in Sudan.^12^

A high prevalence of low FVC (58.5%), which is indicative of reduced lung volumes, was found in the overall population, and women tended to have a higher prevalence of low FVC than men. This finding is lower than the finding from the BOLD study in Ile-Ife, Nigeria (70.4%).^7^ In contrast, this is higher than the findings from a recent study from Tunisia that reported a prevalence of 26%.^34^ The reasons behind this high prevalence are not known but studies suggested that unknown environmental factors, genetics, early exposure to biomass fuels, preterm delivery, malnutrition, childhood respiratory infections, TB and persistent HIV infection may influence lung development and result in lung damage or abnormal lung functioning in such settings.^2^,^7^,^8^,^34^ These factors may have contributed to the high estimates of low FVC in this study.

Exposure to other environmental factors such as living in poor communities may also have been a factor, as 9 of 35 villages were ‘Cambo’. Mostly displaced from underdeveloped and conflicts areas, these populations are among the poorest in Sudan. The association between poverty and lung abnormality has previously been reported in several studies.^9^,^24^

The association between low FVC and being underweight in this study is consistent with previous studies suggesting that being underweight or obese is a risk factor associated with ‘restricted’ lung function.^7^,^34^,^35^

To note, higher estimates of low FVC were identified in those younger than 40 years, whereas participants aged >70 and those who smoked >20 years were less likely to have low FVC. This is in contrast to studies reporting that ageing and smoking were associated with low FVC.^7^,^34^,^35^ It is difficult to explain this finding, but the small number of participants who were >70 years and smoked for more than 20 years could be considered a survivor effect.

### Comparisons with the Urban Khartoum study

Overall, CAO, a key characteristic of COPD was more prevalent in the urban (Khartoum) vs. rural (Gezira) study for participants aged ≥40 years. A higher educational level reported in Gezira State compared to Khartoum State may have contributed to the lower levels of post-BD obstruction observed.^30^ Furthermore, although Gezira State is officially classified as a rural state, it is relatively wealthy as it used to have the largest agriculture project in the country.^17^ Moreover, the high prevalence of CAO in Khartoum might be due to exposure to air pollutants as a result of the large number of factories and cars in the State. Despite a relatively high smoking prevalence, cigarette smoking was not associated with CAO in either study. This might be because respectively 50% and 94% of the smokers reported a smoking history of fewer than 10 pack-years in urban and rural studies.

Rural participants had a lower FVC prevalence estimate and higher respiratory symptoms in the overall population compared to urban ones. We did not explore the reasons for low FVC in this study. Our findings suggest the need for more research in this area. However, early life exposures as a consequence of living in a poor environment, malnutrition and respiratory infections during childhood may have contributed to the high levels of low FVC in this study.^8^

In addition, the higher estimates of low FVC using NHANES III compared to local values suggest that the Sudanese have smaller lungs than Europeans, which is consistent with other studies in Africa.^8^,^25^ Findings from both studies suggest that the burden of low FVC requires more attention and investigation. Airway reversibility in both rural and urban studies highlights the need for further investigation of asthma in the region.

This study is the first of its kind in rural Sudan; the inclusion of a younger age group of ≥18 years allowed us to explore different conditions and lung abnormalities. Spirometry was conducted according to ATS standards with careful quality control. The use of the BOLD standardised method allowed comparability with other findings from the continent and worldwide. This study acknowledges certain limitations, notably a response rate of 56%, with 22% of the initial sample having relocated from the area.

In conclusion, the prevalence of CAO in participants aged ≥40 years, airway reversibility and high prevalence of respiratory symptoms across age groups, especially in younger ones, suggest that CRDs are a serious public health problem in rural Sudanese adults, and highlights the need for more investment in prevention and management of CRDs. The high prevalence of low FVC is one of the key findings in both urban and studies. There is a need for further investigation of the reasons behind the reduced lung volumes in Sudanese adults given its association with increased mortality in other settings.
